# Identification and Functional Characterization of the Leg-Enriched Chemosensory Protein PxylCSP9 in *Plutella xylostella* (Lepidoptera: Plutellidae)

**DOI:** 10.3390/biology14121746

**Published:** 2025-12-05

**Authors:** Shuhui Fu, Fangyuan Li, Xizhong Yan, Chi Hao

**Affiliations:** 1 Department of Biology, Xinzhou Normal University, Xinzhou 034000, China; fushuhui091@163.com; 2 Shanxi Key Laboratory of Integrated Pest Management in Agriculture, Shanxi Agricultural University, Taiyuan 030031, China; 3 College of Plant Protection, Shanxi Agricultural University, Taigu 030800, China

**Keywords:** leg transcriptome, tissue expression, fluorescent competitive binding assays, molecular docking

## Abstract

*Plutella xylostella*, a major pest of cruciferous vegetables, depends predominantly on chemoreception to locate host plants. Legs are crucial in insect chemical perception, particularly during close-range and contact chemoreception. However, the molecular basis underlying the chemosensory repertoire in *P. xylostella* legs remains elusive. To address this, we sequenced chemosensory-related genes in diamondback moth legs. Sequencing identified 32 odorant binding protein (OBP), 18 chemosensory protein (CSP), 26 odorant receptor (OR), 20 gustatory receptor (GR), 15 ionotropic receptor (IR), and 3 sensory neuron membrane protein (SNMP) genes. Comparative analysis with antennal transcriptome data revealed three CSPs, seven ORs, and two GRs newly identified in the legs. Transcriptome analysis showed higher fragments per kilobase of transcript per million mapped reads values for CSPs than for other chemosensory-related gene families. Furthermore, qRT-PCR confirmed the highest expression of *PxylCSP9* in the legs, suggesting its role in perceiving external compounds. Fluorescent binding assays revealed high binding affinity of PxylCSP9 for several host plant semiochemicals. Molecular docking predicted a hydrophobic binding pocket in PxylCSP9 with Met11, Leu13, and Leu43 frequently participating in ligand interactions. Our findings indicate that leg-enriched PxylCSP9 is pivotal for host plant recognition during close-range chemoreception, suggesting its potential as a molecular target for precision management through behavior-based strategies.

## Simple Summary

Phytophagous insects rely on highly evolved chemosensory systems to recognize plant cues, which guide essential behaviors such as feeding and oviposition. While the antennal chemosensory functions of *Plutella xylostella* are well-studied, little is known about the role of its legs in detecting host plant chemicals. This study systematically identified and characterized chemosensory-related genes expressed in the legs of *P. xylostella* using transcriptome data. A total of 114 leg-expressed chemosensory genes were identified, with PxylCSP9 showing high expression in legs and strong, broad-spectrum binding to key cruciferous plant volatiles. Leg chemosensory proteins, particularly PxylCSP9, play an important role in the chemical perception.

## 1. Introduction

Agricultural pests have gradually evolved extremely complex and sensitive chemosensory systems in the process of mutual adaptation and struggle with their host plants [1,2]. These systems dominate several crucial behavioral decisions, such as foraging, courtship, oviposition, and evasion of predators and toxic substances, thereby ensuring insect survival and reproduction [3,4,5]. Host plant selection by insects is a complex and continuous process that involves identifying the host plants from a distance, landing on the plants, close contact evaluation, and ultimately deciding whether to feed on the plants [6,7]. Insects primarily utilize olfactory organs, such as antennae, to locate host plants at a distance, whereas for the close-range evaluation of food, they rely on chemical organs, such as mouthparts and legs [8,9,10]. Several studies have confirmed that some lepidopteran insects, upon landing on the surface of plants, frequently touch the surface of leaves with their legs to perceive the chemical signals emitted by the plants [11,12]. These behaviors enable moths to assess the safety and edibility of potential host plants, and even determine whether the plants are suitable for female adults to lay eggs and for their offspring (larvae) to feed on [6,13,14].

Insect chemosensory functions mainly depend on several functionally interrelated gene families, including odorant binding proteins (OBPs), chemosensory proteins (CSPs), olfactory receptors (ORs), gustatory receptors (GRs), ionotropic receptors (IRs), and sensory neuron membrane proteins (SNMPs) [15,16,17]. OBPs and CSPs in the sensilla lymph can recognize and bind chemical signals from the external environment, and transport them to the corresponding receptors, which is the initial step of the chemosensory system [18]. Chemoreceptors, namely ORs, GRs, and IRs, are activated by external semiochemicals transported by carrier proteins that convert chemical signals into electrical signals, which are transmitted to the neural center for decoding. ORs are responsible for sensing odors [19,20], GRs perceive sugars, amino acids, plant secondary metabolites, and carbon dioxide [21,22,23,24], and IRs combine a variety of functions, including olfactory and gustatory, and play a role in the perception of organic acids and amines, as well as temperature and humidity [25,26,27]. SNMPs are also responsible for the perception of general odors and pheromones, serving an auxiliary role in olfactory recognition [28,29].

These chemosensory-related proteins are indispensable for chemical communication and perception in insect legs. Transcriptome results have revealed that the legs of insects, such as *Apis cerana cerana* (Hymenoptera: Apidae), *Ectropis obliqua* (Lepidoptera: Geometridae), *Apolygus lucorum* (Hemiptera: Miridae), and *Plagiodera versicolora* (Coleoptera: Chrysomelinae), contain OBPs, CSPs, ORs, GRs, IRs, and SNMPs [6,30,31,32]. *A. lucorum* AlucOBP9 is highly expressed in the legs and has a strong ability to bind plant volatiles, suggesting that *A. lucorum* legs are involved in recognizing chemical cues at a close range [33]. Various chemosensory-related proteins in the legs of fruit flies are associated with courtship behavior; for example, GR68a is specifically expressed in the forelegs of male fruit flies and directly participates in the recognition of female sex pheromones [34]. The three chemosensory proteins identified from the leg transcriptome of *A. lucorum* exhibit strong binding abilities with gossypol, a secondary metabolite of its host plants [33]. Thus, chemosensory-related proteins play a crucial role in chemical sensing in insect legs.

The diamondback moth, *Plutella xylostella* L. (Lepidoptera: Plutellidae), is a notorious agricultural pest that specializes in feeding on cruciferous plants worldwide [35,36]. It is difficult to control *P. xylostella* owing to its high reproductive capacity, strong dispersal ability, and rapid development of insecticide resistance. As a member of the Lepidoptera, adult *P. xylostella* of both sexes utilizes sophisticated chemosensory systems to execute critical biological processes for survival and reproduction. In *P. xylostella*, host plant selection relies primarily on the olfactory detection of isothiocyanates, while emerging evidence suggests that post-landing feeding and oviposition behaviors are mediated through antennal and even leg contact with leaf surfaces [20,37,38]. The chemosensory function of the antennae in *P. xylostella* has been extensively investigated; however, research on the role of the legs in chemical communication remains limited. Therefore, to elucidate the leg-mediated molecular mechanisms underlying host location in *P. xylostella*, we identified chemosensory-related genes in the legs using the transcriptomic data. Based on transcriptome analysis and tissue expression profiling, one candidate, *PxylCSP9*, was found to be highly expressed in the legs. The binding affinities of PxylCSP9 to ligands were then confirmed through fluorescence competitive binding assays. Molecular docking were employed to predict the key amino acids of PxylCSP9 involved in ligand binding. This study provides foundational insights into the role of legs in plant–insect interactions.

## 2. Materials and Methods

### 2.1. Insect and Tissue Collection

The *Plutella xylostella* adults used in this study were originally collected from cabbage fields in Taigu District, Shanxi Province, China in 2018, and have been raised as a laboratory colony for over 10 generations. The insects were reared in an artificial climate chamber at 25 ± 1 °C and 75 ± 5% relative humidity with a 16 h light: 8 h dark photoperiod. Larvae were fed fresh cabbage leaves, whereas adults received a 15% (*v*/*v*) honey solution. After eclosion, adult individuals were separated based on sex, and various tissues were collected. For transcriptome sequencing, legs were excised from 100 female and 100 male adults. For qRT-PCR, 300 antennae, 50 heads without antennae, 30 thoraxes, 30 abdomens, 100 legs, and 100 wings were collected from each sex. Three separate biological replicates per tissue sample were used for transcriptome sequencing and qRT-PCR experiments. All tissues were dissected using RNase-free forceps under a stereomicroscope and immediately placed into microcentrifuge tubes placed on a freezing rack floating on liquid nitrogen. The collected samples were stored at −80 °C until use.

### 2.2. cDNA Library Construction and Function Annotation

Total RNA was extracted from different tissue samples using TRIzol reagent (Sangon, Shanghai, China) following the manufacturer’s instructions. The quantity of RNA was determined using a NanoDrop 2000 spectrophotometer (NanoDrop, Wilmington, DE, USA), and its integrity was examined using an Agilent Bioanalyzer model 5400 (Agilent Technologies, Santa Clara, CA, USA). cDNA libraries were constructed using approximately 1.5 μg of RNA template for each sample. The constructed libraries were purified using AMPure XP beads (Beckman Coulter, Beverly, CA, USA) and sequenced on the Illumina NovaSeq 6000 platform.

To ensure the quality and reliability of the data analysis, clean data were obtained by filtering adaptor sequences, poly-N reads (N represents undetermined base information), and low-quality sequences (reads with more than 50% of bases having a Phred quality score < 20). Q20 (indicating percentage of sequences with a sequencing error rate less than 1%), Q30 (indicating a 0.1% error rate), and GC-content of the clean data were calculated simultaneously. Transcriptome assembly was performed using the Trinity software (v2.4.0). The assembly yielded two classes of unigenes: the consensus cluster sequences and singletons. To acquire functional annotations, transcripts longer than 150 bp were subjected to BLASTX and BLASTN homology searches against pooled databases of Non-redundant Protein Sequences (NR), Nucleic Acid Sequences (NT), and SwissProt protein sequences with an E-value of <1 × 10^−5^. BLAST results were further imported into the Blast2GO (b2g4pipe v2.5) pipeline for Gene Ontology (GO) annotation.

### 2.3. Identification of Putative Chemosensory Genes

In addition to keyword searching (e.g., “odorant binding protein”, “chemosensory protein”), a FASTA file of unigenes was created from a local nucleotide database file using BioEdit Sequence Alignment Editor (version 7.1.3.0). Local BLASTN searches were performed against FASTA using the available chemosensory-related genes of *P. xylostella*, *Helicoverpa armigera* (Lepidoptera: Noctuidae), *Bombyx mori* (Lepidoptera: Bombycidae), and *Spodoptera exigua* (Lepidoptera: Noctuidae) as queries, with a threshold E-value of < 1 × 10^−5^. Candidate unigenes encoding putative chemosensory-related genes were manually checked using BLASTX on the NCBI website.

### 2.4. qRT-PCR

The relative expression levels of CSPs in various tissues were detected by qRT-PCR using the Bio-Rad CFX Connect Real-Time Detection System. The protocols used for RNA extraction and cDNA synthesis were the same as those used for the previous cDNA library construction. Ribosomal protein S4 (RPS4) (GenBank: XM_011555372) was used as a reference gene to normalize the expression levels of *CSP* genes. Primers for the target genes and the reference gene were designed using Primer 3 (http://primer3.ut.ee/) (accessed on 26 May 2020) and are listed in the Supplementary Materials Table S1. The specificity of each primer set was validated by melting curve analysis, and amplification efficiency was calculated using standard curves with a five-fold cDNA dilution series. The qRT-PCR was performed in a 20 μL reaction mixture containing 10 μL of 2 × SG Fast qPCR Master Mix (Sangon, Shanghai, China), 0.4 μL of each primer (10 μM), 200 ng of template cDNA, and sterilized ddH_2_O. The thermal cycling conditions were as follows: initial denaturation at 95 °C for 3 min; followed by 40 cycles of denaturation at 95 °C for 10 s, annealing at 58 °C for 10 s, and elongation at 72 °C for 30 s. Each sample was analyzed in three biological replicates, with each biological replicate including three technical replicates. The comparative 2^−ΔΔCt^ method [39] was used to calculate the relative gene expression quantification across tissues. All data analyses were performed using the SPSS Statistics software (version 17.0). Significant differences in expression among different tissues were analyzed using one-way analysis of variance (ANOVA), followed by Tukey’s honestly significant difference test (*p* < 0.05).

### 2.5. Heterologous Expression and Purification of Recombinant PxylCSP9 Protein

The sequence encoding the mature PxylCSP9 protein was amplified by PCR using an upstream primer with a BamHI restriction site and a downstream primer with an Xhol restriction site (Supplementary Materials Table S1). The recovered PCR products were ligated to the pMD19-T (simple) vector (TaKaRa, Dalian, China) and then transformed into *Escherichia coli* DH5α competent cells. The extracted plasmid DNA was digested using BamHI and Xhol restriction enzymes (TaKaRa, Dalian, China). The digested fragments were purified and ligated into the pET-28a(+) expression vector (Novagen, Madison, WI, USA), which was previously linearized using the same restriction enzymes. The recombinant plasmids containing the correct sequences were transformed into *E. coli* BL21(DE3) competent cells. The expression of recombinant PxylCSP9 protein was induced with 0.6 mM isopropyl β-D-1-thiogalactopyranoside (IPTG) at 30 °C for 4 h. Bacterial cells were harvested by centrifugation at 8000× *g* for 10 min, re-suspended in 20 mM Tis-HCl buffer (pH 7.4), and sonicated on ice (15 s pulse duration, 20 passes). After centrifugation at 12,000× *g* for 10 min, the supernatant was collected and purified using Ni-NTA Resin (TransGen Biotech, Beijing, China). The purified recombinant protein was analyzed using 15% sodium dodecyl sulfate-polyacrylamide gel electrophoresis (SDS-PAGE).

### 2.6. Fluorescence Competitive Binding Assays

To identify the ligands that bind to PxylCSP9, we selected several typical chemical components from the host plants of *P. xylostella* for fluorescence competitive binding experiments. The purity of all ligands and the fluorescent probe 4,4′-dianilino-1,1′-binaphthyl-5,5′-sulfonate (Bis-ANS) was ≥95%, which was purchased from Aladdin (Shanghai, China) or Sigma-Aldrich (St. Louis, MO, USA.). All the compounds were dissolved individually in chromatography-grade methanol (Aladdin, Shanghai, China) to prepare 1 mM stock solutions. Fluorescence competitive binding assays were performed using an RF-5301 Fluorescence Spectrophotometer (Shimadzu, Kyoto, Japan) with a quartz cuvette of a 1 cm path length. The excitation and emission slit widths were both set to 5 nm. The excitation wavelength of the fluorescent probe was 365 nm, and the emission spectra were recorded from 400 to 580 nm.

The affinity of Bis-ANS for PxylCSP9 was measured by titrating a 1 μM solution of the protein in 50 mM Tris-HCl (pH 7.4) with aliquots of 1 mM Bis-ANS stock solution to final probe concentrations ranging from 1 to 10 μM. In competitive binding assays, the ligand concentrations ranging from 0 to 10 μM were added to a solution containing PxylCSP9 (1 μM) and Bis-ANS (1 μM). All data were analyzed based on the assumptions that the protein was 100% active and that binding followed a 1:1 stoichiometry (protein:ligand) at saturation. The binding curves for Bis-ANS were linearized using a Scatchard plot. The dissociation constants of the competitors were calculated from their corresponding IC_50_ values using the following equation: K_i_ = [IC_50_]/(1 + [Bis-ANS]/K_Bis-ANS_), where IC_50_ is the ligand concentration that reduces the initial fluorescence intensity of Bis-ANS by half, [Bis-ANS] is the free concentration of Bis-ANS, and K_Bis-ANS_ is the dissociation constant of the PxylCSP9/Bis-ANS complex.

### 2.7. Homology Modeling and Molecular Docking

The homology model of PxylCSP9 was generated using the SWISS-MODEL online server (https://swissmodel.expasy.org/) (accessed on 15 November 2024) based on the structure of *Mamestra Brassicae* (Lepidoptera: Noctuidae) chemosensory protein MbraCSP6 (PDB ID: 1kx9, chain A). Sequence alignment between PxylCSP9 and the template protein was performed using the Clustal X online website (https://www.genome.jp/tools-bin/clustalw) (accessed on 22 November 2024), and the results were visualized with ESPript 3.0 (https://espript.ibcp.fr/ESPript/cgi-bin/ESPript.cgi) (accessed on 22 November 2024). PyMOL (version 2.7.9) software was used to display and analyze the three-dimensional (3D) structures of PxylCSP9 and MbraCSP6. The quality of the constructed 3D structure was evaluated using the SAVES v6.0 structure validation server (https://saves.mbi.ucla.edu/) (accessed on 13 December 2024). Ligand-binding sites within the protein structures were predicted using PrankWeb (https://prankweb.cz/) (accessed on 20 March 2025). The validated model was then prepared for docking, and the Protein Data Bank (PDB) file of PxylCSP9 was converted to a PDBQT file using AutoDock Tools 1.5.6 (ADT), by adding hydrogen atoms and assigning atom types. The 3D structures of ligands exhibiting strong binding affinities in fluorescence competitive binding assays were downloaded from PubChem (https://pubchem.ncbi.nlm.nih.gov/) (accessed on 20 March 2025) and converted to PDBQT format using AutoDock Tools 1.5.6 (ADT). Molecular docking of PxylCSP9 with selected ligands was performed using AutoDock Vina 1.1.2, with the docking grid center defined at coordinates x = −7.783, y = 29.326, z = 26.862. The nine top-ranked conformations for each ligand were obtained, each providing information on the binding affinity and pose conformation. The results of the interaction between PxylCSP9 and the ligands were analyzed and visualized using Discovery Studio 4.5 Client (Accelrys, San Diego, CA, USA).

## 3. Results

### 3.1. Overview of the Leg Transcriptome

**Figure 1:**
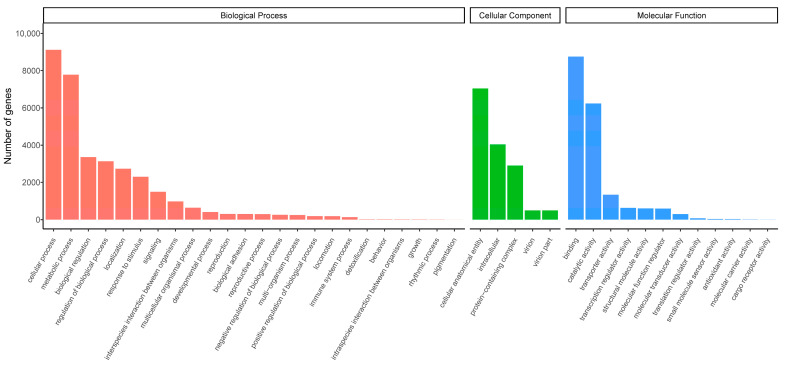
Gene Ontology functional annotations of unigenes in the leg transcriptome of *Plutella xylostella*.

Transcriptome datasets were successfully obtained from six leg samples of female and male *P. xylostella*. The data volume of each sample exceeded 6 GB. The GC content was not less than 45.87%, Q20 was greater than 96.30%, and Q30 was greater than 90.38%, indicating high-quality sequencing data suitable for subsequent assembly (Supplementary Materials Table S2). The clean reads were assembled into 141,226 transcripts. Further assembly of these transcripts produced 46,554 unigenes, with a total length of 51,410,982 bp, an average length of 1104 bp, and an N50 length of 1791 bp (Supplementary Materials Table S2). All unigenes were blasted against seven public databases (NR, NT, KEGG, SwissProt, PFAM, GO, and KOG) for functional annotations. A total of 35,314 unigenes (75.85%) were functionally annotated in at least one database. Among them, the NT database annotated the most unigenes (31,074; 66.74%), whereas the KOG database annotated the least (8552; 18.37%). With the GO classification, 16,189 unigenes were categorized into three functional classes: biological processes, cellular components, and molecular functions. In the biological processes class, cellular processes were the most abundant. In the cellular component class, the cellular anatomical entities were the most common. In the molecular function class, binding-related genes were the most highly represented subclass (Figure 1).

### 3.2. Identification of Putative Chemosensory-Related Genes in P. xylostella Legs

In total, 114 chemosensory-related genes were identified in the leg transcriptome of *P. xylostella*. These genes encoded 32 OBPs (including two GOBPs and three PBPs), 18 CSPs, 26 ORs (including one ORco), 20 GRs, 15 IRs (including four co-receptors: IR8a, IR25a, IR76b, and IR93a), and three SNMPs (Table 1). Notably, three CSPs, seven ORs, and two GRs were newly identified. Each identified OBP sequence contained a complete open reading frame (ORF) and a signal peptide at the N-terminus, exhibiting six conserved cysteine residues arranged in the classical pattern C_1_-X_26–36_-C_2_-X_3_-C_3_-X_37–42_-C_4_-X_6–12_-C_5_-X_8_-C_6_. All of 18 CSPs possessed full-length ORFs with a signal peptide at the N-terminus and four conserved cysteine residues, which are typical characteristics of insect CSPs. Although 26 candidate ORs, 20 candidate GRs, and 15 IRs were identified in the legs, the number of these genes was lower than that reported in the antenna transcriptome. Three candidates of PxylSNMPs possessed full-length ORFs exceeding 522 amino acids in length.

### 3.3. Expression Profiles of Chemosensory-Related Genes in P. xylostella Legs

**Figure 2:**
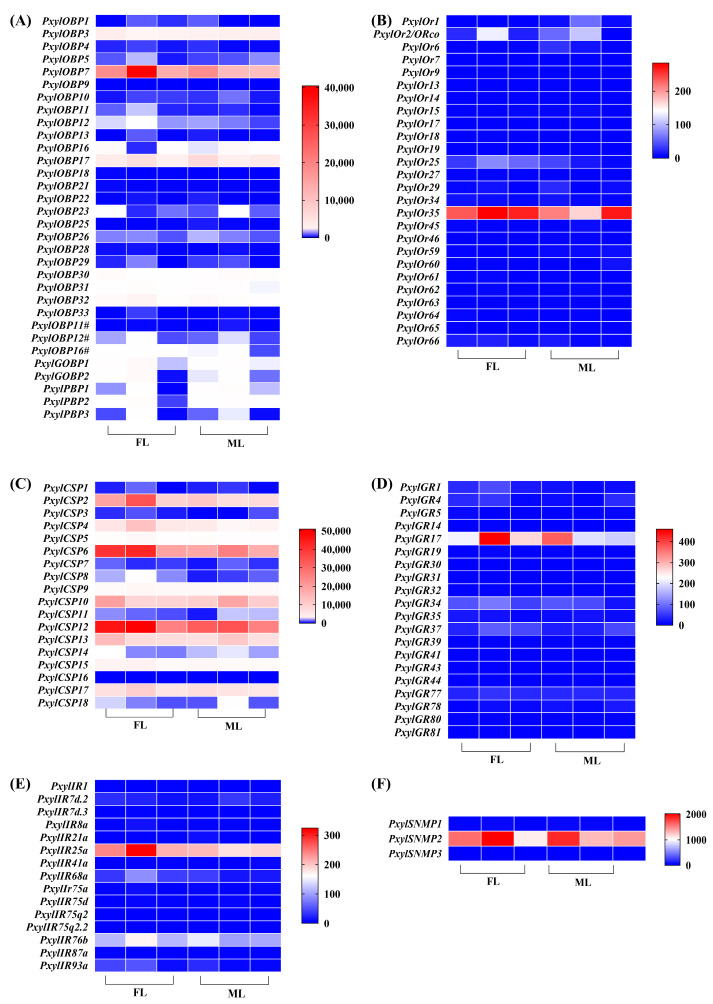
Heatmaps of chemosensory-related genes of *P*. *xylostella* based on fragments per kilobase of transcript per million mapped reads (FPKM) values. (**A**–**F**) OBPs, ORs, CSPs, GRs, IRs and SNMPs, respectively. FL: female adult legs; ML: male adult legs.

The expression levels of chemosensory-related genes in *P. xylostella* legs were analyzed based on fragments per kilobase of transcript per million mapped reads (FPKM) values from transcriptome sequencing. Overall, *P. xylostella* legs showed higher expression levels of *OBP* and *CSP* genes than other chemosensory-related genes (*OR*, *GR*, *IR,* and *SNMP* genes) (Figure 2). Among the *OBP* genes, the expression levels of *PyxylOBP3*, *PyxylOBP7*, and *PyxylOBP17* were higher in the legs of both male and female adults, with *PyxylOBP7* showing the highest expression. Ten *CSP* genes (including *PxylCSP2*, *PxylCSP4-6*, *PxylCSP9-10*, *PxylCSP12-13*, *PxylCSP15*, and *PxylCSP17*) exhibited high expression levels in the legs of *P. xylostella*, of which *PxylCSP12* showed the highest expression. *PxylOR35* showed the highest expression among *ORs*, *PxylGR17* among *GRs*, *PxylIR25a/PxylIR76b* among *IRs*, and *PxylSNMP2* among *SNMPs* in the legs of both male and female *P. xylostella*.

### 3.4. Tissue Expression Patterns of PxylCSPs

**Figure 3:**
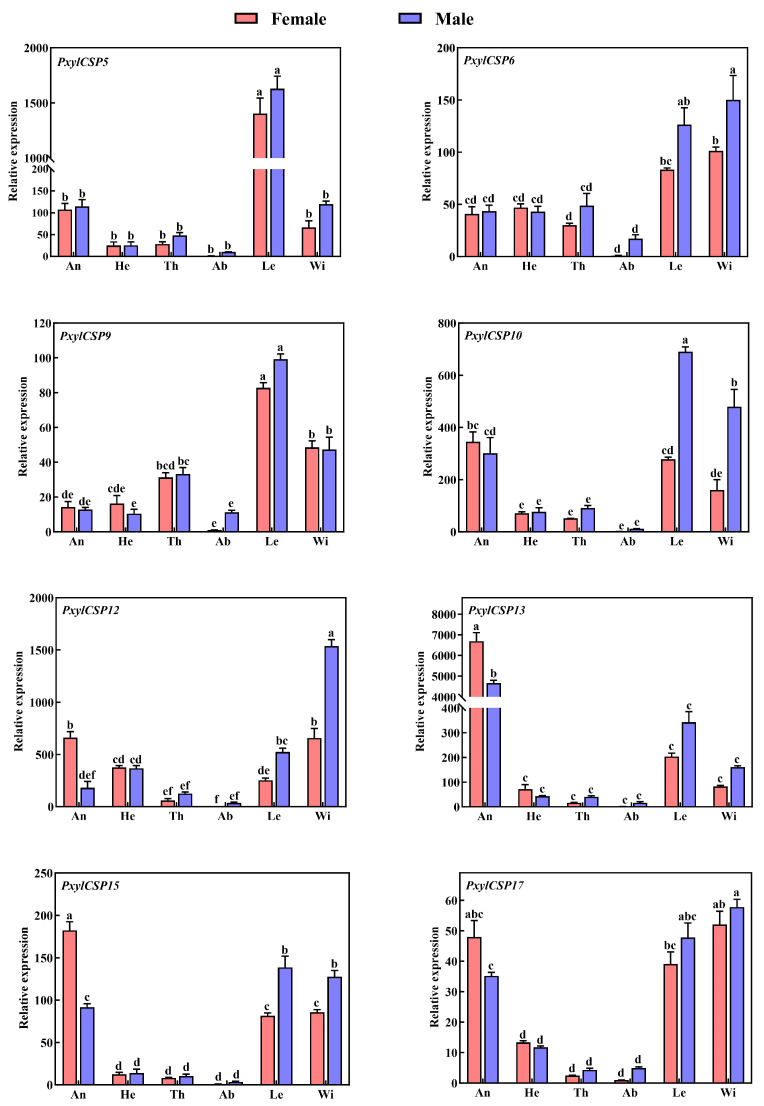
Tissue expression levels of CSPs in *P*. *xylostella.* An, antennae; He, heads without antennae; Th, thoraxes; Ab, abdomens; Le, legs; Wi, wings. Different lowercase letters above the bars indicate significant differences (*p* < 0.05) among tissues.

Given the high FPKM values of some PxylCSPs, we performed qRT-PCR to determine their tissue expression profiles and screen for candidate genes with high expression in the legs. The assessed genes were expressed in various tissues, including the antennae, heads without antennae, legs, wings, and other body tissues of both sexes, but exhibited distinct expression patterns (Figure 3). *PxylCSP13*, *PxylCSP15,* and *PxylCSP17* were highly expressed in the antennae of both sexes, with significantly higher *PxylCSP13* expression in the antennae than in other tissues. *PxylCSP15* and *PxylCSP17* displayed relatively high expression levels in not only the antennae but also in the legs and wings. *PxylCSP5*, *PxylCSP9*, and *PxylCSP10* showed higher expression levels in the legs than in the other tissues in both sexes. Furthermore, *PxylCSP5* and *PxylCSP9* were expressed in the legs of females and males, showing no significant differences between the sexes. *PxylCSP6* and *PxylCSP12* were highly expressed in the wings of both females and males.

### 3.5. Cloning, Heterologous Expression, and Purification of PxylCSP9

Because of the high FPKM value and enriched expression of *PxylCSP9* in the legs of *P. xylstella* among all CSP genes, we selected it for cloning and functional analysis. The full-length cDNA sequence of *PxylCSP9* was 372 bp, encoding 123 amino acid residues and containing four conserved cysteine residues. The polypeptide contained a predicted 17-residue signal peptide at its N-terminus. Upon removal of the signal peptide, the mature PxylCSP9 protein had a molecular weight of 12.57 kDa and an isoelectric point of 8.35.

**Figure 4:**
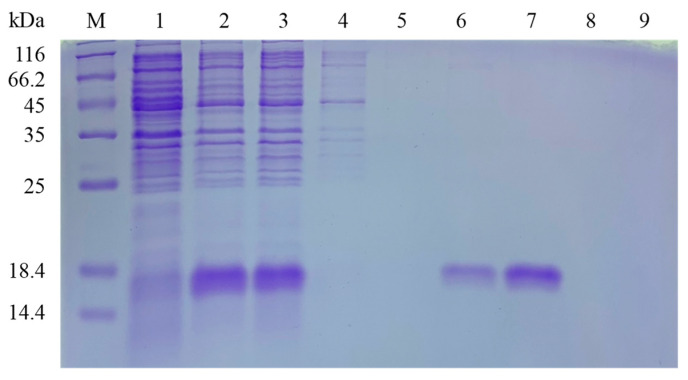
SDS-PAGE analysis of purified PxylCSP9 recombinant protein. M, Protein molecular weight standard; 1: expressed products of BL21-pET28a(+)-PxylCSP9 without induction by IPTG; 2: expressed products of BL21-pET28a(+)-PxylCSP9 with induction by 1 mM IPTG; 3: supernatant; 4–9: elution solution with 10, 20, 50, 100, 250, and 500 mM imidazole buffer, respectively.

After cloning the PxylCSP9 cDNA into the pET28a(+) prokaryotic expression vector, the successful expression of PxylCSP9 recombinant proteins in *E. coli* BL21(DE3) competent cells was confirmed by 15% SDS-PAGE. The induced recombinant PxylCSP9 was predominant in the supernatant after bacterial lysis by ultrasonication. The target protein was purified using ProteinIso Ni-NTA resin and subsequently eluted in large amounts with 50 and 100 mM imidazole buffers (Figure 4). The purified recombinant protein was used in subsequent experiments.

### 3.6. Characterization of PxylCSP9 Bound to Odorants

**Figure 5:**
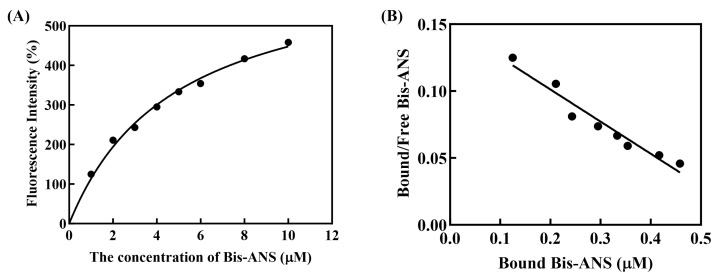
Binding curve of PxylCSP9 to Bis-ANS and relative Scatchard plot analysis. (**A**) Bind curve of PxylCSP9 to Bis-ANS; black dots represent the actual measured fluorescence intensity value at each specific Bis-ANS concentration, the solid line is the computed binding curves. (**B**) Scatchard plot of PxylCSP9 to Bis-ANS. The black circles represent the bound/free ratio of Bis-ANS against its bound concentration, with values calculated from the measured fluorescence intensity. The solid line is generated by linear fitting of these points.

In the competitive binding assays with host plant volatiles, the fluorescent probe Bis-ANS was a suitable probe for PxylCSP9, as evidenced by a characteristic gradually increase in fluorescence intensity (Figure 5A). A linear Scatchard plot (Figure 5B) confirmed binding, with a calculated dissociation constant of 4.86 ± 0.04 μM, supporting its use in subsequent experiments.

**Figure 6:**
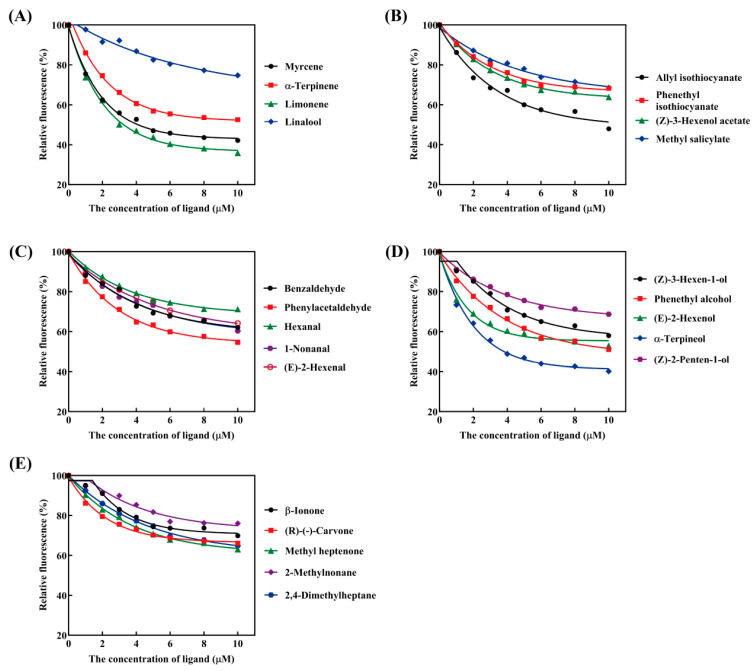
Competitive binding curves of PxylCSP9 with different volatile ligands. (**A**), Monoterpenes; (**B**), Esters; (**C**), Aldehydes; (**D**), Alcohols; (**E**), Ketones and alkanes.

PxylCSP9 exhibited remarkable binding capabilities towards all tested host plant volatiles, indicating a diverse and extensive binding spectrum. PxylCSP9 exhibited strong affinity (Ki ≤ 10 µM) for limonene (Ki = 3.12 µM), α-terpineol (Ki = 3.80 µM), myrcene (Ki = 4.15 µM), α-terpinene (Ki = 7.68 µM), allyl isothiocyanate (Ki = 8.12 µM), and phenethyl alcohol (Ki = 8.34 µM) (Figure 6 and Table 2). Except for 2-methylnonane (Ki = 30.83), PxylCSP9 displayed moderate affinity (10 < K_i_ ≤ 30 μM) for the other volatiles, with K_i_ values ranging between 10.08 μM and 29.19 μM (Figure 6; Table 2).

### 3.7. Modeling and Molecular Docking of PxylCSP9

**Figure 7:**
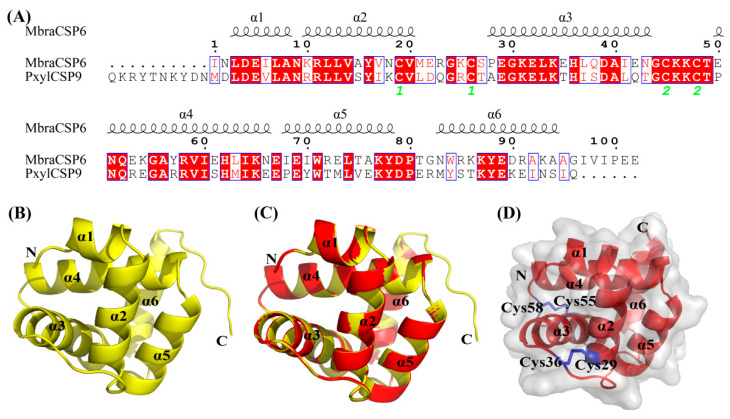
Three-dimensional (3D) structures of PxylCSP9 from *P. xylostalla.* (**A**) Sequence alignment of *P. xylostalla* PxylCSP9 and homologous protein MbraCSPA6. Identical residues are highlighted in red, similar amino acids are boxed in blue, conserved cysteine residues forming disulfide bonds are numbered in green, and α-helices are indicated by black helical lines. (**B**) 3D structure of the template MbraCSPA6. (**C**) Superimposed structure of PxylCSP9 and the template MbraCSPA6. (**D**) 3D structure of PxylCSP9. N and C represented the amino terminal and carboxyl terminal of the protein, respectively; α indicated α-helix; Cys was cysteine.

The three-dimensional model of PxylCSP9 was constructed using MbraCSPA6 (ID: 1kx9.2.A) from *M. brassicae* as a template, which shared 54.81% high sequence identity. The model comprised six α-helices (α1–α6) and two disulfide bridges (Cys29-Cys36 and Cys55-Cys58), which were consistent with typical structural features of insect CSPs (Figure 7).

**Figure 8:**
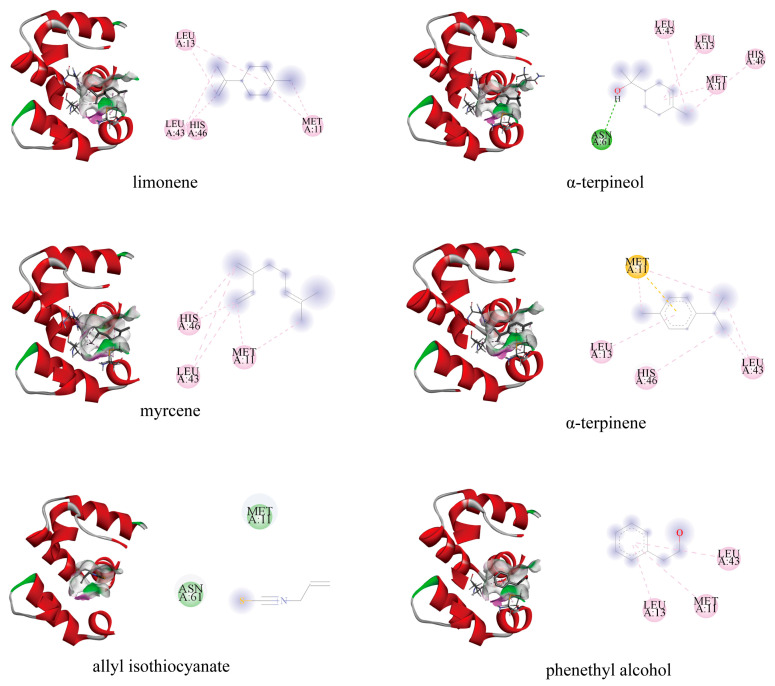
Molecular docking analysis of the binding characters about PxylCSP9 with ligands. Green, pink, and orange dashed lines indicate hydrogen bonds, hydrophobic interactions, and π-sulfur interaction, respectively. Green circles without dashed lines represent the amino acid with van der Waals forces between PxylCSP9 and the ligand.

The amino acid residues Met11, Leu13, Val16, Glu42, Leu43, His46, Asn61, Gln62, Gly65, and Ala66 of PxylCSP9 collectively formed a hydrophobic binding pocket (Figure 8). All the ligands were located in the hydrophobic pocket. The binding energies ranged from −3.0 to −4.8 kcal/mol. The interaction between PxylCSP9 and its ligands was mediated by various polar and non-polar bonds. Asn61 of PxylCSP9 formed a hydrogen bond (H bond) with α-terpineol. Met11, Leu13, and Leu43 were also the crucial amino acid residues and appeared frequently among all the tested compounds, indicating their importance in binding to various host plant volatiles.

## 4. Discussion

Insect antennae have been widely recognized as the primary organs for volatile chemical perception, and emerging evidence has revealed enriched chemosensory-related genes in other appendages, particularly in the leg tarsus [32,52,53]. To systematically investigate the molecular mechanisms underlying chemosensory perception in the legs of *P. xylostella*, we identified and analyzed chemosensory-related genes using high-throughput transcriptome sequencing technology.

In the present study, a total of 114 candidate chemosensory-related genes, including 32 OBPs, 18 CSPs, 26 ORs, 20 GRs, 15 IRs, and three SNMPs, were identified from the transcriptome of *P. xylostella* legs. Yang et al. [41] identified 118 chemosensory-related genes (24 OBPs, 15 CSPs, 54 ORs, seven GRs, 16 IRs, and two SNMPs) using antenna transcriptome data. Comparative analysis of antennal transcriptome datasets demonstrated a reduced chemosensory-related gene repertoire in leg tissues relative to the antennae in *P. xylostella*. This is in line with the findings of previous studies on *P. versicolora* and other insects, such as the identification of 98 chemosensory-related genes in the antennae of *P. versicolora*, as opposed to only 53 in the legs [32]. Comparative analysis of quantitative differences in the distribution of chemosensory-related gene families between the antennae and legs of *P. xylostella* revealed that OBPs, CSPs, GRs, and SNMPs were more abundant in the legs, with GRs being significantly enriched, whereas ORs and IRs were less abundant in the legs, with ORs exhibiting a pronounced reduction. This phenomenon reflects the functional differentiation of insect sensory organs [54]: antennae are specialized in long-range olfactory detection, while legs are involved in contact chemosensation and mechanoreception. Notably, most chemosensory-related genes identified in the legs of *P. xylostella* were also present in its antennae, suggesting their potential role in chemical perception within the legs.

FPKM values calculated from RNA sequencing (RNA-seq) data are commonly used to evaluate the expression levels of candidate genes [55]. Transcriptome analysis revealed that FPKM values of OBPs and CSPs were significantly higher than those of other chemosensory genes (ORs, GRs, IRs, and SNMPs) in the legs of *P. xylostella*. Notably, the number of *CSP* genes (10 members: *PxylCSP2*, *PxylCSP4*-*6*, *PxylCSP9*-*10*, *PxylCSP12*-*13*, *PxylCSP15*, and *PxylCSP17*) with high FPKM values exceeded that of the OBPs (four members: *PxylOBP3*, *PxylOBP7*, *PxylOBP17*, and *PxylOBP32*), suggesting their predominant role in the legs. Given their robust expression, we focused on elucidating the functional significance of CSPs in legs. The tissue-specific expression patterns of *CSP* genes in insects are typically correlated with their particular physiological functions [56,57]. Here, we analyzed the tissue-specific expression patterns of CSP-encoding genes in *P. xylostella* across male and female adults using qRT-PCR. To avoid redundancy, two *CSP* genes (*PxylCSP2* and *PxylCSP4*) with well-characterized tissue expression patterns in previous studies (e.g., Liu et al. [58]) were omitted from the qRT-PCR experiments. *PxylCSP* genes were predominantly distributed in the antennae, legs, and wings, suggesting their functional diversity. CSPs highly expressed in the antennae are related to olfactory recognition and involved in host localization, oviposition selection, and mate search. *PxylCSP13*, *PxylCSP15*, and *PxylCSP17* were exclusively or highly expressed in female and male antennae of *P. xylostella,* which may be related to olfactory recognition. The expression levels of *PxylCSP9* and *PxylCSP10* were higher in the legs than in other tissues. Moreover, *PxylCSP9* was specifically expressed in the legs of both sexes, with no significant difference in expression levels between females and males, whereas *PxylCSP10* showed significantly higher expression in the legs of male insects than females. Sex-biased differences in gene expression patterns are associated with distinct functions in males and females. Thus, PxylCSP9 may be related to close perception of host plants, and PxylCSP10 may be involved in courtship behavior. Currently, insect CSPs are believed to be involved in diverse tasks from behavior to several different physiological and biological processes beyond chemoreception [18]. *PxylCSP6* and *PxylCSP12* were abundant in the wings of both sexes. The functions of these PxylCSPs need to be further characterized.

To further elucidate the potential roles of the CSPs enriched in the legs, we selected PxylCSP9 as the target protein to assess its ligand affinity for various host plant volatiles. PxylCSP9 displayed distinct binding affinity towards all tested chemicals, suggesting that PxylCSP9 has a rather broad and diverse ligand-binding ability. Of all ligands, PxylCSP9 exhibited the strongest binding ability towards six host plant volatiles, namely limonene, α-terpineol, myrcene, α-terpinene, phenylethyl alcohol, and allyl isothiocyanate. Allyl isothiocyanate, a member of the isothiocyanate compounds, is a specific volatile found in the cruciferous plants [59]. This volatiles can induce feeding and oviposition behaviors in *P. xylostella* [37,47]. Phenylethyl alcohol, a common floral scent compound frequently found in various plants, including cruciferous species, can elicit electroantennogram (EAG) responses and attractive behaviors in certain lepidopteran insects [48,60,61]. Limonene, α-terpinene, and myrcene, as monoterpene volatiles released by cruciferous vegetables, effectively stimulate the EAG response of *P. xylostella*, but significantly induce its behavioral avoidance response [49,51,62]. Furthermore, limonene and α-terpinene specifically exhibit oviposition deterrent effects [49]. The five aforementioned volatiles can be divided into two categories based on their behavioral effects on the diamondback moth: (1) attractants, which “pull” the diamondback moth to approach the host plants, and (2) repellents, which “push” the moth away from the plants. The binding affinities of PxylCSP9 to attractants and repellents suggest that it may acts as a broad-spectrum signal transducer, transmitting “attractive” and “repellent” signals to the central nervous system, prompting the system to determine the final behavioral output. Therefore, PxylCSP9 may be involved in the moth’s searching for host plants, and locating oviposition sites.

The 3D modeling and docking tests were used to further analyze the specific binding properties of PxylCSP9. The binding affinity of the ligand depends on the specific positioning of the amino acids within the hydrophobic region of the protein [63]. We found that Met11, Leu13, Val16, Glu42, Leu43, His46, Asn61, Gln62, Gly65, and Ala66 collectively formed a hydrophobic binding pocket in PxylCSP9, which is crucial for mediating protein-ligand interactions. Notably, Asn61 formed a hydrogen bond (H bond) with α-terpineol, suggesting that polar interactions may complement the predominantly hydrophobic binding environment. Among the residues analyzed, three hydrophobic amino acids, Met11, Leu13, and Leu43, appeared most frequently in the docking simulations, indicating their essential roles in anchoring ligands within the binding pocket. These residues stabilize ligand interactions primarily through van der Waals forces and hydrophobic interactions. Similar amino acids and interactions have been observed in the binding of other insect chemosensory proteins to ligands. For example, leucine and isoleucine in *M. brassicae* MbraCSPA6 and *Bactrocera minax* (Diptera: Tephritidae) BminCSP3 [64] participate in the binding of chemosensory proteins to ligands via hydrophobic interactions. Our findings indicate the dual contribution of hydrophobic interactions and specific hydrogen bonding to ligand recognition by PxylCSP9, with Met11, Leu13, and Leu43 serving as key residues for binding affinity and selectivity. Further investigations, such as site-directed mutagenesis or structural studies, will help elucidate the functional contributions of these key residues to the ligand-binding mechanism of PxylCSP9.

Our study, employing an integrated approach including transcriptomics, qRT-PCR, fluorescent competitive binding assays, and molecular docking, revealed that PxylCSP9 is highly expressed in the legs of *P. xylostella* and exhibits a broad binding spectrum to host plant volatiles, thus implicating its potential role in the chemosensory processes of the moth’s legs. However, these findings are primarily derived from in vitro experiments. Future research utilizing RNA interference (RNAi) or gene knockout techniques, such as CRISPR/Cas9, coupled with behavioral assays, will be essential to directly verify the physiological function of PxylCSP9 in vivo and to assess its potential as a target for sustainable pest management.

## 5. Conclusions

In this study, we successfully identified 114 chemosensory-related genes in the legs of *P. xylostella*, encompassing 32 OBPs, 18 CSPs, 26 ORs, 20 GRs, 15 IRs, and 3 SNMPs. Transcriptome analysis showed that *CSP* genes had higher FPKM values than other chemosensory-related gene families in the legs. Further qRT-PCR results revealed that *PxylCSP9* was predominantly expressed in the legs compared to other tissues. Fluorescent competitive binding assays showed that PxylCSP9 had high affinities to several host plant volatiles, including limonene, α-terpineol, myrcene, α-terpinene, allyl isothiocyanate, and phenethyl alcohol, indicating its role in mediating the localization of *P. xylostella* to host plants. This study not only deepens our understanding of insect chemical sensing mechanisms but also provides new molecular targets for the green control of diamondback moth.

## Figures and Tables

**Table 1:** Number of chemosensory-related genes in the leg transcriptome of *Plutella xylostella*.

Chemosensory-Related Genes	Number of Genes in the Legs	Number of Newly Found Genes	Number of Reported Genes
Odorant binding protein (OBP) genes	32	0	39 [40,41]
Chemosensory protein (CSP) genes	18	3	15 [41]
Odorant receptor (OR) genes	26	7	59 [20]
Gustatory receptor (GR) genes	20	2	67 [42]
Ionotropic receptor (IR) genes	15	0	36 [43]
Sensory neuron membrane protein (SNMP) genes	3	0	3 [41,44]

**Table 2:** Affinities of PxylCSP9 with different volatile ligands.

Ligands	IC_50_ (μM)	K_i_ (μM)
Monoterpenes		
Myrcene ^b^	4.92 ± 0.65	4.15
α-Terpinene ^b^	9.10 ± 0.31	7.68
Limonene ^b^	3.70 ± 0.13	3.12
Linalool ^b^	30.89 ± 8.71	26.07
Esters		
Allyl isothiocyanate ^a^	9.62 ± 0.58	8.12
Phenethyl Isothiocyanate	27.98 ± 1.53	23.61
(Z)-3-Hexenol acetate ^a^	21.02 ± 0.65	17.73
Methyl salicylate	31.85 ± 4.23	26.88
Aldehydes		
Benzaldehyde	18.97 ± 1.64	16.01
Phenylacetaldehyde	11.98 ± 0.72	10.11
Hexanal ^a^	33.21 ± 1.34	28.04
1-Nonanal ^a^	18.82 ± 1.16	15.88
(E)-2-Hexenal ^a^	21.49 ± 0.41	18.10
Alcohols		
(Z)-2-Penten-1-ol	30.79 ± 3.94	25.95
(Z)-3-Hexen-1-ol ^a^	13.90 ± 0.32	11.73
Phenethyl alcohol ^a^	9.90 ± 0.43	8.34
(E)-2-Hexenol ^a^	11.97 ± 0.91	10.08
α-Terpineol	4.49 ± 0.14	3.80
Ketones		
β-Ionone	26.51 ± 2.53	22.37
(R)-(-)-Carvone ^b^	34.64 ± 6.62	29.19
Methyl heptenone	20.10 ± 1.98	16.96
Alkanes		
2,4-Dimethylheptane ^b^	20.62 ± 0.46	17.40
2-Methylnonane ^b^	36.53 ± 4.30	30.83

^a^ Refers to plant volatiles that are attractive to *P. xylostella* [37,45,46,47,48]. ^b^ Refers to plant volatiles that are repellent to *P. xylostella* [49,50,51].

## Data Availability

The raw data of RNA-Seq were uploaded to the NCBI database under the accession number NCBI Bioproject PRJNA1330265.

## Supplementary Materials

The following supporting information can be downloaded at: https://www.mdpi.com/article/10.3390/biology14121746/s1, Figure S1: Overall quality factor of PxylCSP9 model evaluated by ERRAT; Figure S2: Ramachandran plot and statistics of PxylCSP9 model analyzed by PROCHECK; Table S1: All primer pairs in this study; The capital letters in italics were the protective bases of restriction endonucleases, the underlined letters were restriction endonucleases, the restriction endonuclease of the upstream primer was BamHI, and the restriction endonuclease of the downstream primer was XhoI. Table S2: Results of the quality of sequencing data from the legs of adult *Plutella xylostella;* Table S3: Statistics of sequence length of the transcriptome of legs in *P. xylostella.* Table S4: Detailed information of candidate ligand compounds in fluorescence competitive binding experiments.

## Abbreviations

The following abbreviations are used in this manuscript:

OBPs	Odorant binding proteins
CSPs	Chemosensory proteins
ORs	Olfactory receptors
GRs	Gustatory receptors
IRs	Ionotropic receptors
SNMPs	Sensory neuron membrane proteins
FPKM	Fragments per kilobase of transcript per million mapped reads
IPTG	Isopropyl β-D-1-thiogalactopyranoside
ORF	Open reading frame
