# Supplementary material for: Identification and Functional Characterization of the Leg-Enriched Chemosensory Protein PxylCSP9 in Plutella xylostella (Lepidoptera: Plutellidae)
Source: Biology (Basel). 2025 Dec 5;14(12):1746. doi: 10.3390/biology14121746 (PMC12730722; doi:10.3390/biology14121746)
Supplement: Supplementary file 1 [file biology-14-01746-s001.zip › biology-3994657-supplementary.pdf]

Table S1. All primer pairs in this study

Primer name	Primer sequence (5’ to 3’)
For qRT-PCR analysis	
PxylCSP5-F	AAGTACGACAACATCAACCT
PxylCSP5-R	CTTCTCCAGCACACAGTT
PxylCSP6-F	GCACGGAGAAGCAGAAGTCA
PxylCSP6-R	CCTGGTAGATGTTGTTGGGG
PxylCSP9-F	TAAGACACACATCAGCGACG
PxylCSP9-R	CCTCTCAGGGTCGTACTTCTC
PxylCSP10-F	GCTGGTGCCCTACATCAAAT
PxylCSP10-R	GTATTCCGTCTCCTTCTCTCTGA
PxylCSP12-F	GATGTTGGTAGTGGCTGCG
PxylCSP12-R	CAGGGTCCCTTGTCGATCAG
PxylCSP13-F	TGTGACGACATACAGAGCCA
PxylCSP13-R	CGTACTTGGTGGTGAACTGC
PxylCSP15-F	AGCCGCTTAGAGACAGTC
PxylCSP15-R	TACTTCTCACGCTGCTTC
PxylCSP17-F	GCTGATGACGACACATTCTCC
PxylCSP17-R	AGCCTTCACTGACTTCCTTACA
RPS4-F	ATGGATGTTGTGTCGATTGAAAAGA
RPS4-R	GGGGTTGCCCAGGTCAGAT
Expression vector construction	
PxylCSP9	*CGC*GGATCCCAGAAGCGGTACACCAA<br>*CCG*CTCGAGCTGGATACTATTGATCTCCTT

The capital letters in italics were the protective bases of restriction endonucleases, the underlined letters were restriction endonucleases, the restriction endonuclease of the upstream primer was *Bam*HI, and the restriction endonuclease of the downstream primer was *Xho*I.

Table S2. Results of the quality of sequencing data from the legs of adult *Plutella xylostella*.

Sample	Raw reads	Clean reads	Clean bases (GB)	Q20 Percentage (%)	Q30 percentage (%)	GC percentage (%)
Female 1	23 882 529	23 319 356	7	96.48	90.97	50.39
Female 2	25 593 363	24 973 569	7.5	96.62	91.07	47.56
Female 3	20 453 758	20 062 095	6	96.66	91.18	47.97
Male 1	23 588 804	23 035 693	6.9	96.91	91.62	46.85
Male 2	21 479 399	21 094 740	6.3	96.30	90.38	47.97
Male 3	22 054 949	21 772 828	6.5	96.40	90.52	45.87

Table S3. Statistics of sequence length of the transcriptome of legs in *P. xylostella.*

	N50 (bp)	N90 (bp)	Minimum length (bp)	Maximal length (bp)	Number	Mean length (bp)
Contigs	1,707	499	301	20,755	141,226	1,175
Unigenes	1,791	440	301	20,755	46,554	1,104

Table S4. Detailed information of candidate ligand compounds in fluorescence competitive binding experiments.

Compounds	CAS numbers	Compounds	CAS numbers
Myrcene	123-35-3	1-Nonanal	124-19-6
α-Terpinene	99-86-5	(E)-2-Hexenal	592-43-8
Limonene	5989-54-8	(Z)-2-Penten-1-ol	1576-95-0
γ-Terpinene	99-85-4	(Z)-3-Hexen-1-ol	928-96-1
Linalool	78-70-6	Phenethyl alcohol	60-12-8
Allyl isothiocyanate	57-06-7	(E)-2-Hexenol	13419-69-7
Phenethyl Isothiocyanate	2257-09-2	α-Terpineol	98-55-5
(Z)-3-Hexenol acetate	3681-71-8	β-Ionone	14901-07-6
Methyl salicylate	119-36-8	(R)-(-)-Carvone	99-49-0
Benzaldehyde	100-52-7	Methyl heptenone	110-93-0
Phenylacetaldehyde	122-78-1	2,4-Dimethylheptane	2213-23-2
Hexanal	66-25-1	2-Methylnonane	871-83-0

Figure S1. Overall quality factor of PxylCSP9 model evaluated by ERRAT

Program: ERRAT2
File: PxylCSP9.pdb
Chain#:A
Overall quality factor**: 98.851

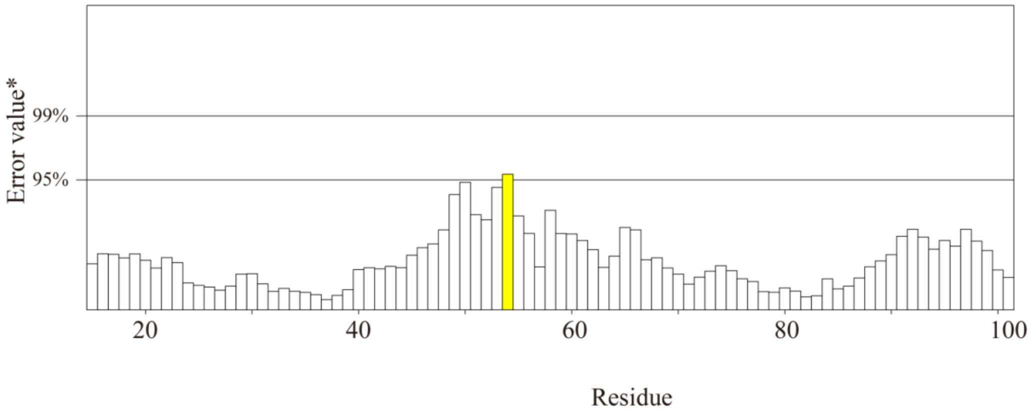

*On the error axis, two lines are drawn to indicate the confidence with which it is possible to reject regions that exceed that error value.

**Expressed as the percentage of the protein for which the calculated error value falls below the 95% rejection limit. Good high resolution structures generally produce values around 95% or higher. For lower resolutions (2.5 to 3A) the average overall quality factor is around 91%.

Figure S2. Ramachandran plot and statistics of PxylCSP9 model analyzed by PROCHECK

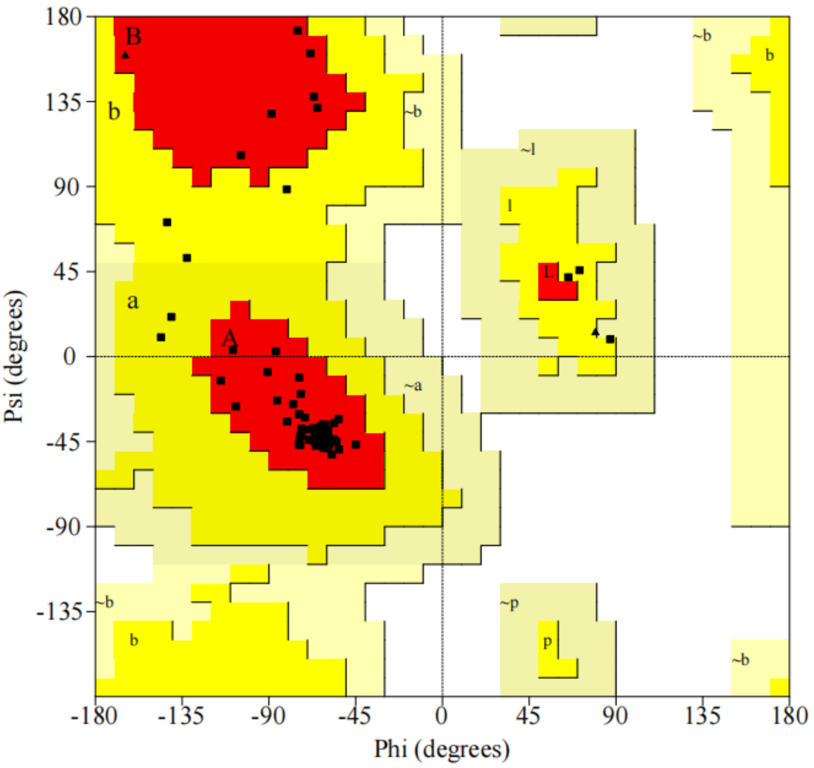

Plot statistics

Residues in most favoured regions [A,B,L]	78	90.7%
Residues in additional allowed regions [a,b,l,p]	8	9.3%
Residues in generously allowed regions [~a,~b,~l,~p]	0	0.0%
Residues in disallowed regions	0	0.0%
	----	------
Number of non-glycine and non-proline residues	86	100.0%
Number of end-residues (excl. Gly and Pro)	2	
Number of glycine residues (shown as triangles)	4	
Number of proline residues	3	

Total number of residues	95	

Based on an analysis of 118 structures of resolution of at least 2.0 Angstroms and R-factor no greater than 20%, a good quality model would be expected to have over 90% in the most favoured regions.
